# Integrating the Genomic Revolution into Newborn Screening: Current Challenges and Future Perspectives

**DOI:** 10.3390/pediatric18010014

**Published:** 2026-01-19

**Authors:** Albina Tummolo, Emanuela Ponzi, Simonetta Simonetti, Mattia Gentile

**Affiliations:** 1 Department of Metabolic Diseases and Clinical Genetics, Giovanni XXIII Children Hospital, Azienda Ospedaliero-Universitaria Consorziale, 70126 Bari, Italy; 2 Department of Medical Genetics, Di Venere Hospital, 70012 Bari, Italy; emanuela.ponzi@asl.bari.it (E.P.); mattia.gentile@asl.bari.it (M.G.); 3 Department of Clinical Pathology and Neonatal Screening, Children’s Hospital “Giovanni XXIII”, Azienda Ospedaliero-Universitaria Consorziale, 70126 Bari, Italy; simonetta.simonetti@policlinico.ba.it

**Keywords:** newborn screening, tandem mass spectrometry, next-generation sequencing, multiomics, metabolic disorders, genomics

## Abstract

In recent years, the development of new diagnostic technologies, such as tandem mass spectrometry (MS/MS) and next-generation sequencing (NGS), has caused a veritable revolution in the diagnosis of genetic diseases, reducing time, cost, and invasiveness associated with prior diagnostic techniques. While MS/MS laid the foundation for the development of numerous, usually institutionally based, neonatal screening programs, NGS has gained traction in newborn screening (NBS), primarily through pilot projects and private funding across different countries. As a result, the traditional Wilson and Jungner criteria have been supplemented by additional criteria, including considerations of equity and access, in response to emerging technologies. This review aims to provide an up-to-date overview of the global landscape of metabolic screening panels, highlight the major ongoing genomic screening projects, and outline the current models for integrating these two screening systems. Substantial differences exist across countries in the numbers and types of diseases included in national NBS programmes. In this context, Italy represents a prominent case, as its neonatal screening framework has seen significant expansion and development in recent years, reaching a particularly comprehensive metabolic screening panel. Nonetheless, a number of initiatives to incorporate genomic technologies into the NBS pathway are currently underway, primarily involving high-income countries. Nonetheless, unlike metabolomic-based NBS programs, no country has a government-mandated NGS program as first-tier testing for newborns. New evidence is emerging from ongoing models of integration of multi-omics approaches into NBS, including the use of AI and machine learning. Identifying the most appropriate system for this integration to reduce the false-positive and false-negative rates associated with both screening types, ensure more equitable access to screening, and facilitate faster access to treatment may represent a useful and foresightful way to conceptualize NBS in the future. This transitional phase should promote rigorous improvements before full-scale adoption.

## 1. Introduction

Newborn screening (NBS) has been a cornerstone in paediatric health care, allowing the early detection of different conditions and enabling timely interventions to improve outcomes [1,2]. This practice began more than half a century ago with one condition, the inherited metabolic disorder (IMD) Phenylketonuria (PKU). In this case, the heel prick test, first introduced by Robert Guthrie [3], enabled babies with PKU to be identified soon after birth, determining significant changes in PKU natural history [4,5,6,7,8].

Since the 1990s, a key technique for NBS has been represented by tandem mass spectrometry (MS/MS), which provides a method for the simultaneous identification of numerous metabolites with diverse chemical profiles within a single analytical assay. This technique allowed the reduction of costs associated with expanding screening compared to the traditional one test–one condition model [9,10,11]. Since then, the number of conditions included in neonatal screening programs has increased considerably over time [12,13,14,15].

Over the last decade, we have witnessed a further significant technical revolution in the diagnosis of genetic conditions [16,17,18]. Following the established success of MS/MS, the advent of DNA sequencing technologies has marked a new turning point in medicine, enabling the reading, analysis, and interpretation of the human genome and achieving definitive diagnoses in increasingly shorter times with the introduction of next-generation sequencing (NGS) [19,20,21]. Gradually, the idea of integrating genetic testing into neonatal screening as a first-tier test emerged and was implemented at varying speeds across countries [22,23].

As a result, the traditional Wilson and Jungner criteria [24] have been supplemented by additional criteria, including considerations of equity and access, in response to emerging technologies, helping to address unmet medical needs and reduce population health inequities [25,26]. Nowadays, NGS applied to NBS is increasingly recognised, but strategies for integrating metabolomic and genomic-based screening results are not yet defined or standardised.

This review aims to provide an overview of the most up-to-date NBS situation worldwide, illustrate the ongoing projects related to genome-based NBS, and provide an overview of the proposed model of integration between metabolomic- and genomic-based NBS.

## 2. Metabolomic-Based Newborn Screening: The Current Worldwide Situation

MS/MS-based multiplexing, profiling amino acids and acylcarnitines from a single DBS, facilitated the inclusion of various organic acidurias, fatty acid oxidation defects, and amino acidemias into routine programs, providing rapid diagnostic results while remaining cost-effective [27]. There are marked differences in NBS composition across European nations, with some countries using wide screening panels and others narrow panels, made up of no more than two conditions or not screening any IMDs yet [28]. In Italy, NBS is considered an essential public health service and became mandatory by law in 1992 when only three disorders were screened for [29]. In 2016, new rare diseases were added, covering one of the largest screening panels worldwide. More recently, in November 2025, another eight treatable, genetically determined disorders were added to the NBS panel by extending the essential assistance levels [30]. The screening panel undergoes periodic regular revision and an annual report, provided by the national scientific society for NBS and IMDs [31].

As of November 2025, the NBS program in France covers 16 conditions and has undergone a significant expansion since 1 September 2025. The French health technical agency recommended extending the NBS programme in line with other European countries, including Spinal Muscular Atrophy (SMA) and Adenosine Deaminase Deficiency—Severe Combined Immunodeficiency (ADA-SCID) [32,33,34]. Germany has also expanded the screening panel over the years, and at present, the panel includes 19 diseases. In addition, regional pilot studies on additional diseases are conducted to evaluate feasibility, diagnostic process quality, and potential health benefits of an extended panel [35,36].

In Belgium, the panel reportedly includes 16–20 treatable metabolic defects detected by tandem mass spectrometry [37,38]. The UK’s National Health Service screens for a relatively smaller core set of conditions compared to many other high-income countries. Currently (as of late 2025), it reports 10 core conditions, including Tyrosinaemia type 1 (HT1), recently added in October 2025 [39,40]. The Netherlands has one of the most comprehensive NBS panels in Europe. It currently reports a total of 27 rare disorders, including Adrenoleukodystrophy (ALD) and Mucopolysaccharidosis type 1 (MPS I) [41,42].

Southeastern Europe comprises 14 countries, 7 of which are EU member states. NBS programmes in the region are generally underdeveloped, with only Hungary, Croatia, and Slovenia actively using MS/MS for NBS detecting. Some countries in the region have NBS programmes limited to a few disorders [28,43,44]. Greece have recently introduced NBS via MS/MS for 29 disorders, Cyprus only screens for two disorders, and in some cases, such as Albania, there is no NBS programme available for the population [45].

In the United States, most states follow the Recommended Uniform Screening Panel (RUSP), which recommends screening for a core panel of 35 conditions and 26 secondary conditions [46]. States may adopt the entire RUSP or choose a subset. Some states, such as New York, are testing 50 or more conditions in their standard panel, supplementing RUSP conditions with other rare diseases [47,48]. Each individual state designs, manages, and funds its own screening programme, meaning that the number of diseases screened can vary significantly by state of birth [49,50].

In Asia, coverage and panels vary [51,52]. Although accounting for about half of the world’s births, NBS coverage remains significantly low on average [53]. Some countries, such as Japan [54,55,56], Taiwan [57,58,59,60], Singapore [61], Hong Kong [62,63], Australia [64,65,66], and New Zealand [67,68], have well-established and universal newborn screening programmes, with coverage rates exceeding 99 per cent. In other Asian developing countries, the highest proportions of populations have limited access to programmes, often based on regional and private initiatives [69,70]. Coverage in countries such as India [71,72] or Indonesia [73,74] remains very low. China has a minimum national panel, but regions can add to it, with coverage generally being high (>90%) [75,76]. India, despite its large population, has not yet implemented an active national NBS programme [77,78].

The NBS situation in Latin America is highly heterogeneous and, although it has made significant progress in many countries, it still poses challenges in terms of coverage, uniformity, and number of screened pathologies [79,80,81]. Approximately 15 out of 20 countries have NBS programmes [82,83], but the disorders screened for and the overall coverage vary widely, remaining below 70% in some countries, such as Mexico [84,85]. The vast majority perform an NBS for PKU and congenital hyperthyroidism [86,87]. Brazil screens for six IMDs and is working to expand its panel to include other conditions, such as lysosomal diseases and SMA [88,89,90,91]. Argentina and Colombia screen for six and eight IMDs, respectively; they are planning to include other conditions using MS/MS as the detection technology [92,93,94].

The situation of newborn screening in African countries, particularly sub-Saharan Africa, is characterized by extremely low coverage and, in many cases, the absence of universal screening programs at the national level [95], with less than 1 million babies screened annually out of approximately 30 million births [96,97]. South Africa has programs that, although not universally uniform, include traditional screening for congenital hypothyroidism and PKU in some provinces and sometimes also cystic fibrosis and sickle cell anaemia in high-risk areas [98,99]. Nigeria has limited NBS programmes for sickle cell anaemia, confined to specialised centres or research projects in specific cities [100,101]. Egypt has a more established program [102,103].

## 3. Genome-Based Newborn Screening

The Human Genome Project, completed in 2003, paved the way to modern genomics and enabled the identification of genetic variants associated with numerous genetically determined diseases, including those with neonatal onset [104]. Moreover, it fostered the development of increasingly sophisticated technologies, which have recently been applied to NBS programs [105,106]. Unlike metabolomic-based NBS programs, no country has a government-mandated NGS program as first-tier testing for newborns. Research initiatives are currently underway in several countries to evaluate the validity, utility, and efficiency of genomics-based neonatal screening technologies [107,108]. Some of these initiatives enrol patients within a single clinical site, while others focus on multiple sites within a single region or across different regions within their countries [109,110,111]. Table 1 reports the main NBS programmes and genomic-based NBS initiatives worldwide, grouped by geographical area of interest.

The first and largest study of genomic newborn screening is GUARDIAN (Genomic Uniform Screening Against Rare Diseases in All Newborns), which began in 2022 in New York City with the goal of sequencing 100,000 newborns [112]. GUARDIAN aims to analyse 255 genetic conditions that typically present in the first five years of life; of these, 156 have well-established treatments. The study includes an optional screening for the other 99 neurodevelopmental disorders with associated conditions for which, although a recognized therapy is not yet available, early interventions may improve outcomes following early diagnosis. Testing was successfully completed for 99.6% of cases. The screen-positive rate was 3.7%, including treatable conditions that are not currently included in NBS [113].

In 2021, the United Kingdom (UK) government funded a program to screen 100,000 newborns for conditions to be treated very early. A list of more than 200 conditions to screen for, with about 460 genes on the panel, has been identified. This programme, called Generation Study, aims to use umbilical cord blood as the sample source [114]. A consortium for NBS in Europe, called Screen4Care, is being developed, involving groups from Italy, France, the Netherlands, Germany, and Denmark [115,116]. At the moment, 14 European countries are members of this consortium. The characteristics of this project will be addressed in the paragraph Neonatal Genomic Screening: Active Projects in Italy.

Apart from this pan-European research study, there are many studies in place within countries and regions, including Germany and Italy, as well as Australia and Belgium.

Babyscreen+ in Australia [117] was designed to enrol a limited number of infants in the State of Victoria, with informed parental consent. The project was supported by the Australian Government’s Medical Research Future Fund. Approximately 500 treatable genetic conditions have been included in the panel. BabyDetect is an NGS genomic program targeting specific genes and was mainly developed in southern Belgium [118]. It screens between 120 and 165 rare and treatable infantile diseases, focusing specifically on diseases that are treatable with early onset. It is not always free: at some stages, it was charged to parents, with the possibility of partial reimbursement from some insurance policies. Large-scale projects are emerging in Asia, with a strong focus on building specific population databases, including the China Neonatal Genomes Project (CNGP) and projects similar to BabySeq currently underway in Taiwan [119,120] (Table 1).

**Table 1:** Main NBS programmes and genomic-based NBS initiatives worldwide.

Continent/Country	Newborn Screening	Key Characteristics	Genomic Screening	Specific Geographical Area	Key Characteristics
**USA**	Traditional NBS (RUSP)	Mandatory, state-funded screening for a Recommended Uniform Screening Panel (RUSP), currently covering over 35 core disorders.	BabySeq [16,17,18], BeginNGS [121], GUARDIAN [112,113]	Boston, San Diego, New York	Studies exploring the clinical utility and ethical implications of whole-genome/exome sequencing (WGS/WES) in newborns
**Australia**	Expanded NBS	Metabolic (biochemical) and DNA.	BabyScreen+ [117]	Victoria State	Large research study assessing the feasibility of adding genomic sequencing (500+ conditions) to the heel-prick test.
**Western Europe**	National/regional programs	Comprehensive programs covering 30–50+ disorders using MS/MS and targeted DNA as second-tier test.	Screen4Care [115,116,122,123]	Italy, France, Netherlands, Germany, Denmark, etc.	Major European initiative aiming to integrate genomic data into NBS for early diagnosis of rare diseases (RDs).
			The Generation Study [114]	UK	Large-scale, genomic research project integrating WGS into the NHS structure (700+ conditions).
			BabyDetect [118]	Belgium	A complementary program using targeted NGS to screen for 100+ conditions, exceeding the standard national panel.
			Italian Regional Projects: RING-Lombardia [124]	Lombardia Region	Explores clinical and organizational aspects of genomic NBS, focusing on three WGS scenarios.
			Genoma Puglia Program [125]	Puglia Region	First publicly funded, structural regional program offering WES/targeted sequencing for ~400 rare diseases.
**ASIA**	Varies widely	Some nations have established programs; others are gradually expanding but often lack uniformity.	China Neonatal Genomes Project (CNGP) [119]	China	Aims to sequence 100,000 newborns over 5 years to create a Chinese reference genetic database.
			Taiwan BabySeq Initiative (TBSI) [120]	Taiwan	Collaboration (started in 2024) to launch a pilot program similar to the American BabySeq.

## 4. Neonatal Genomic Screening: Active Projects in Italy

In Italy, several pilot initiatives on genomic-based NBS are laying the groundwork for its future introduction, with the purpose of a genetic diagnosis of rare diseases from the earliest days of life. One of the most relevant initiatives in this field is Screen4Care, a European project involving several Italian centres and many European countries [122]. The project employs panels of about 245 genes associated with treatable early-onset rare diseases. In Italy, neonatal recruitment began in January 2025, aiming to assess not only technical feasibility but also clinical and organisational impact. To achieve this, Screen4Care has developed two main genetic panels. The first, the TREAT panel, includes approximately 245 genes associated with treatable rare diseases [123]. This panel will be tested in a European pilot study involving around 20,000 newborns. The second, the ACT panel, covers “actionable” rare diseases, conditions for which therapeutic interventions exist, in collaboration with European Reference Networks (ERNs), such as MetabERN and EURO-NMD (European Reference Network for Neuromuscular Diseases). For newborns testing negative on both panels but later showing symptoms suggestive of a genetic disorder, whole-genome sequencing (WGS) will serve as a complementary diagnostic approach to detect conditions not covered by the predefined panels.

Alongside this, some Italian regions have launched autonomous initiatives. A significant initiative is the RINGS Project in Lombardy, which is designed to explore the clinical, ethical, and organisational implications of large-scale genomic neonatal screening [124]. The project is based on three scenarios: (1) the use of WGS as a screening tool for a defined set of conditions; (2) an extended screening approach with no pre-defined condition limits, encompassing all potentially disease-associated variants; and (3) diagnostic use of WGS for newborns with complex clinical presentations lacking a definitive diagnosis. Rather than a direct experimental trial, RINGS focused on developing technical recommendations, outlining potential implementation pathways, and contributing valuable insights to the national debate.

The Apulia Region, with a regional law approved in 2023 [125], established the “Genoma-Puglia” program. This project uses a panel of 407 genes for extended neonatal screening, allowing early detection of over 470–490 genetic diseases. The pilot phase plans to genetically screen about 3000 newborns, with the goal of gradually extending the initiative to the entire regional neonatal population on a voluntary basis. This represents the first example in Italy of a regional legal framework institutionalising neonatal genomic screening.

## 5. Existing Models of Multi-Omics Integration in Newborn Screening

Genetic testing is performed as a second-tier test for most conditions currently included in NBS programs and is used to confirm a diagnosis and to inform the genetic risk of other family members. At present, only a few standard NBS assays measure DNA as an analyte. These include molecular screening for SMA and SCID, which have been introduced into Australia’s and Italy’s NBS programs over the last 5 years [20,126].

A review of the omics literature by Zhuang et al. [127] found that many studies have been performed on DBS using either metabolomic, genomic, epigenomic, or proteomic techniques, but very few have used DBS for multi-omics investigations [128]. One example comes from Kerhofs et al. [129]. They performed untargeted metabolomics on DBS samples from 97 patients with 46 different Inherited Errors of Metabolism (IEMs) to generate a prioritised list of possible disease-causing genes, with variants from whole-exome sequencing subsequently prioritised and interpreted. Similarly, Almeida et al. [130] used an integrated multi-omics approach of first-tier gene panel sequencing with biochemical testing to improve the diagnostic rate of IEMs in blood and DBS samples.

In an NBS setting, an integrated multi-omics approach combining both metabolomic and genomic techniques on DBS samples may overcome the challenges of using either first-tier genomic or metabolomic screening alone, as they are both characterized by specific limitations due to the high incidence of false positives in the metabolomic NBS and the low predictive value of genomic NBS alone.

In a cross-sectional analysis conducted between 2014 and 2019, Liu et al. [131] compared the screening capabilities of traditional metabolic tests with those of a metabolomic screening approach that integrated sequencing and clinical data. They found that metabolomic screening had a sixfold higher diagnostic rate for IEMs than traditional metabolic and biochemical screening and identified a broader range of IEMs.

In the Italian Region of Campania, a workflow-based parallel biochemical and sequencing analyses for NBS has recently been tested to determine whether this approach could improve the diagnostic outcomes of neonates [132]. The advantage of this approach was performing molecular analysis only in positive newborns and using a restricted panel of 105 genes relevant to the metabolomic-based NBS. This allowed a 100% diagnosis rate and a potential reduction in costs related to NBS procedures, reducing the impact on patients and families as it allowed reduced unnecessary hospital access and minimized family anxiety by eliminating the wait times due to false positives, common in traditional screening.

In a recent study, Yu et al. [133] analysed four NBS modes using newborn genomic sequencing and traditional biochemical screening. The combination of both screening procedures was the most satisfactory screening mode, with a detection rate of 99.17%, specificity and positive predictive value of 100%, and negative predictive value of 99.89%.

A cohort study included newborns who were prospectively recruited from eight screening centres in China using DBS [134]. The screen included biochemical screening tests and targeted gene panel sequencing for 128 conditions. Gene panel sequencing identified 59 patients who were undetected by biochemical tests, including 20 patients with biochemically and genetically screened disorders. In this study, the use of gene panel sequencing in a general newborn population as a first-tier screening test improved the detection capability of traditional screening, providing evidence-based support for considering it a crucial method for first-tier screening.

Novel algorithmic approaches, such as machine learning, are being developed to master the high-dimensional complexity of multi-omics NBS data. By integrating genomic, metabolomic, and proteomic layers, these models identify non-linear correlations that traditional methods miss, significantly reducing false positives through better risk stratification. Ultimately, this shift toward precision diagnostics enables personalized screening profiles that may facilitate earlier intervention and more tailored therapeutic strategies for rare genetic disorders [135,136].

There are several AI-driven initiatives in NBS programs globally, aimed at improving the accuracy, speed, and efficiency of detecting rare diseases. CLIR (Collaborative Laboratory Integrated Reports) [137] was developed by Mayo Clinic to enhance metabolic screening in medicine and genomic sequencing accuracy. It uses AI-driven analytics genome sequencing to reduce false positives and flag high-risk cases [138]. The BeginNGS Initiative in the USA combines rapid whole-genome sequencing with AI for real-time variant interpretation. It screens more than 400 genetic diseases with actionable treatment [121].

**Figure 1:**
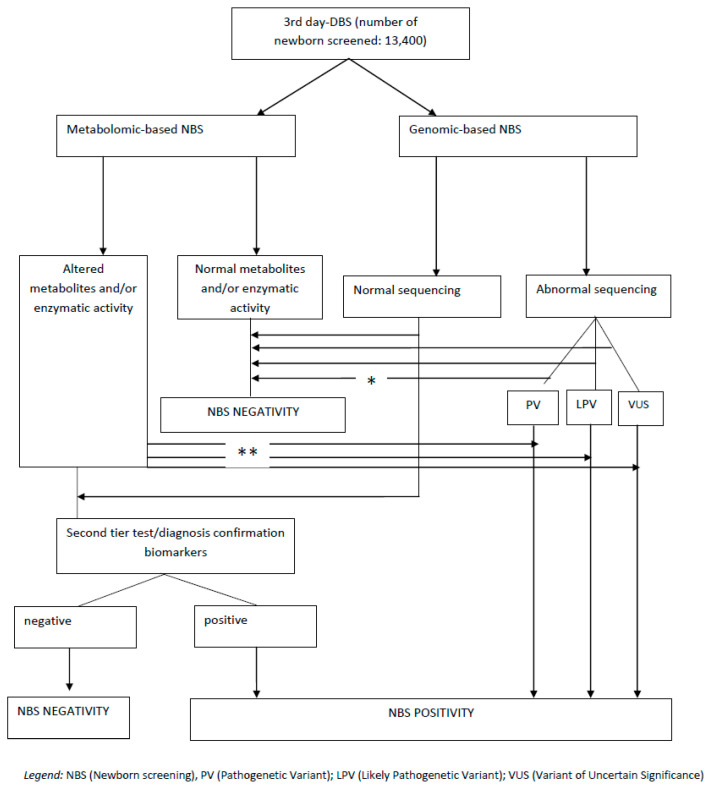
Workflow of integration between metabolomic-based and genomic-based NBS in the Apulia Region. * In cases where metabolomic-based NBS results are negative but genomic screening identifies a PV, the result is categorized as Genomic-Positive/Metabolic-Negative, requiring specialized longitudinal monitoring to detect late-onset, mild, or low-penetrance conditions that are undetectable by standard metabolic screening at birth. ** In cases of positivity for an IMD with high risk for decompensation, this path will be prioritized and completed within 72–96 h from DBS arrival at the laboratory.

The integration model of the Apulia Region involves performing DBS for both metabolic and genomic screening. Figure 1 shows the workflow starting from a single DBS obtained at 36–72 h of life: in this model, genomic NBS acts as the co-first-tier test as it is performed at the same time and regardless of the results of the metabolic one. If the level of screening metabolites or enzyme activity is normal, confirmation of negativity from genomic screening is expected. If both agree, newborn screening is closed as negative.

In the case of a positive metabolic screening, two situations are configured: if an IMD is at high risk of acute decompensation, the genetics laboratory is contacted with top priority, and genetic confirmation will be available within a maximum of 96 h from DBS arrival at the laboratory. In cases with low or no risk of acute decompensation, the patient is recalled to the reference clinical centre, and confirmatory biochemical diagnostic tests are initiated. Once genetic confirmation is obtained, the screening is considered positive.

If metabolic screening is negative, the genetics laboratory is consulted. In cases of pathogenic variant (PV) detection, a biochemical investigation of second-tier markers involved in the metabolic pathway is performed. If there are variants that are likely pathogenic (LPV) or variants of uncertain significance (VUS) in the absence of biochemical alterations and/or normal enzymatic activity, the screening is concluded as negative. In the case where there is negative metabolic NBS but genomic screening identifies a PV, the newborn is categorized as having a Genomic-Positive/Metabolic-Negative status. This scenario triggers a specialized clinical follow-up, which includes longitudinal biochemical monitoring and a specialist evaluation to rule out late-onset phenotypes, reduced penetrance, or mild clinical presentations that may not be detectable via traditional metabolic markers at birth.

If metabolic screening is positive but no gene alteration is identified, patient follow-up is initiated based on the existing metabolic alterations, and the genomic panel is extended to SNP array and whole-genome sequencing (WGS). The newborn is categorized as having a Metabolic-Positive/Genomic-Negative status.

## 6. Discussion

There are large differences between countries in the numbers and types of diseases included in national NBS programmes [139]. Some countries screen for over 30 diseases as part of their national panel, with Italy leading the way in Europe, screening for more than 50 diseases by national law [29]. In contrast, many countries are still currently screening for significantly fewer diseases, and many of them, particularly in Asia, Africa, Latin America, and Southeast Europe, do not screen for any genetic condition [46].

Metabolomic NBS is still the most widely used method for screening newborns worldwide [140], but a number of initiatives to incorporate genomic technologies into the NBS pathway are in the planning stage or have just been implemented, mostly focusing on local and/or regional projects.

**Figure 2:**
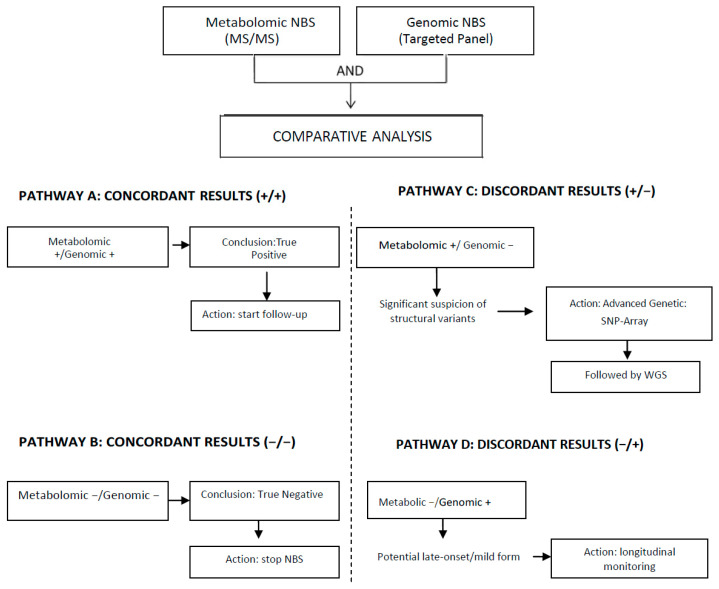
A proposed multi-omics approach to newborn screening.

Compared to genomic testing, the metabolome shows a closer correlation with the phenotype and may provide a more precise indication of disease states, as it integrates genetic variation, gene expression, protein interactions, and regulatory processes [105,113]. On the other hand, genomic testing, unlike traditional biochemical screening, allows analysis of a broader range of genetic conditions, including many rare diseases without metabolic markers that thus escape conventional screening [110,111,112]. It may provide a definitive genetic diagnosis at birth, thus reducing healthcare costs associated with the lengthy diagnostic process. Metabolomics acts as a functional check for genomic findings, confirming if a DNA mutation is actually causing biological effects and genomics acts as a confirmatory tool for metabolomic findings, rapidly identifying the specific genetic cause of a biochemical imbalance. In the model proposed, genomic-based NBS can be considered as co-first-tier test as it is applied on the same DBS and at the same time of the metabolomic NBS, regardless of its result. Figure 2 reports a proposed multi-omics NBS workflow.

The interpretation of whole-genome data remains characterised by many challenges: some variants, although known to cause a recognised disease in childhood, might only manifest later in life, or, in some cases, not at all. Additionally, sequencing the whole genome of newborn babies, in case of genome-based NBS negativity, also allows for identifying possible genetic changes of unknown significance [141,142]. In this context, although well established in a diagnostic setting, with symptomatic newborns, the analytical and clinical validity, sensitivity, and specificity of genome sequencing have not been extensively examined in a screening context, particularly in healthy newborns [143,144].

Beyond technical and interpretative challenges, the widespread implementation of genomic NBS faces significant structural and economic challenges. A primary concern involves the turnaround time for results; while some initiatives like the Apulia Region model aim for rapid confirmation within 96 h for high-risk conditions, larger consortium frameworks such as Screen4Care must address the risk that timelines spanning from several days to 2–3 months may be too late for diseases requiring immediate clinical intervention. Furthermore, the transition toward a genome-first or integrated model necessitates a substantial expansion of the specialist workforce. There is an urgent need for a sufficient number of pediatricians and medical genetic specialists to provide timely counseling to families facing unclear diagnoses or conditions where only actionable treatments are available, ensuring that the ‘genomic revolution’ does not exacerbate existing healthcare inequities.

Despite the above issues, a larger-scale application of genomic neonatal screening may be viable, as many studies highlight the potential benefits of expanding screening programs by implementing this new approach. A re-evaluation of the Wilson and Jungner criteria to fit a multi-omics landscape, standardised protocols, and equitable access must be ensured for this technology to deliver authentic public health and social benefits [145].

New evidence from the ongoing model of integration of genomic and metabolic-based NBS, as well as the use of AI and machine learning to allow a continuously updated combination, emphasize that using biochemical and genomic NBS in parallel may increase the sensitivity of the screening and more newborns may be identified, decreasing the number of false positives and false negative cases, and may represent a useful and foresightful way to conceptualize NBS in the future.

The integration of genomic and multi-omics technologies into NBS pathways threatens to exacerbate existing global health disparities. This transition marks a shift from low-cost metabolic testing to high-complexity diagnostics, creating a significant economic barrier. To prevent a permanent genomic disparity, the world economy must proactively address these inequities. It is imperative to develop international frameworks and funding models aimed at limiting these disparities, ensuring that the benefits of the genomic revolution in neonatal care do not become a privilege reserved to a few nations. This transitional phase may allow for rigorous validation of genomic sensitivity while providing the time needed to resolve complex issues related to variant interpretation, data infrastructure, and access to follow-up and management for people from diverse backgrounds before full-scale adoption.

Further research is imperative to address the multifaceted ethical, legal, and psychosocial challenges linked to future NBS. Specifically, critical attention must be directed toward the management of incidental findings, the ‘right not to know,’ the complexities of data reanalysis, and the mitigation of systemic inequities. Furthermore, refining consent models and implementing robust health economic modeling, including cost–benefit and sustainability analyses, is essential to provide a solid base required for public health adoption and long-term policy integration.

## 7. Conclusions

The transition toward integrated multi-omics models of NBS is not just a technical upgrade but a paramount shift of the NBS concept that requires a structured global roadmap to prevent a permanent division among countries. High-income countries should promote the transition from pilot studies to integrated government programs, leading the development of bioinformatics platforms that can improve NBS result interpretation across countries. Middle- and low-income countries should implement targeted pilot programs focusing on locally highly prevalent and treatable conditions, as well as implementing accessible MS/MS technologies, with support from international funding and training programs by scientific societies. A core set of genetic disorders, where early intervention can significantly modify disease progression, should be prioritized, and global collaboration and training of specialized personnel should be implemented to ensure that the genomic revolution really promotes global health achievement.

## Data Availability

The data that support the findings of this study are available on request from the corresponding author (A.T.).
