# Correction: Tummolo et al. Integrating the Genomic Revolution into Newborn Screening: Current Challenges and Future Perspectives. *Pediatr. Rep.* 2026, *18*, 14

**DOI:** 10.3390/pediatric18020039

**Published:** 2026-03-06

**Authors:** Albina Tummolo, Emanuela Ponzi, Simonetta Simonetti, Mattia Gentile

**Affiliations:** 1 Department of Metabolic Diseases and Clinical Genetics, Giovanni XXIII Children Hospital, Azienda Ospedaliero-Universitaria Consorziale, 70126 Bari, Italy; 2 Department of Medical Genetics, Di Venere Hospital, 70012 Bari, Italy; emanuela.ponzi@asl.bari.it (E.P.); mattia.gentile@asl.bari.it (M.G.); 3 Department of Clinical Pathology and Neonatal Screening, Children’s Hospital “Giovanni XXIII”, Azienda Ospedaliero-Universitaria Consorziale, 70126 Bari, Italy; simonetta.simonetti@policlinico.ba.it

In this paper [1], the authors would like to replace Reference 118 with the correct one that directly refers to the peer reviewed study of the Baby Detect program. The correct Reference 118 is:

118. Boemer, F.; Hovhannesyan, K.; Piazzon, F.; Minner, F.; Mni, M.; Jacquemin, V.; Mashhadizadeh, D.; Benmhammed, N.; Bours, V.; Jacquinet, A.; et al. Population-based, first-tier genomic newborn screening in the maternity ward. *Nat. Med*. **2025**, *31*, 1339–1350.

The authors state that the scientific conclusions are unaffected. This correction was approved by the Academic Editor. The original publication has also been updated.

## References

[1] Tummolo A., Ponzi E., Simonetti S., Gentile M. (2026). Integrating the Genomic Revolution into Newborn Screening: Current Challenges and Future Perspectives. Pediatr. Rep..

